# Filum terminale transection in pediatric tethered cord syndrome: a single center, population-based, cohort study of 95 cases

**DOI:** 10.1007/s00701-022-05218-6

**Published:** 2022-04-28

**Authors:** Erik Edström, Charlotte Wesslén, Alexander Fletcher-Sandersjöö, Adrian Elmi-Terander, Ulrika Sandvik

**Affiliations:** Department of Neurosurgery, Karolinska University Hospital and Department of Clinical Neuroscience, Karolinska Institutet, Stockholm, Sweden

**Keywords:** Spinal cord, Filum terminale, Fatty filum, Tethered cord, Urinary dysfunction, Children

## Abstract

**Purpose:**

The purpose of this study was to evaluate outcome following surgical transection of filum terminale (FT) in symptomatic and asymptomatic pediatric patients with radiological findings consistent with tethered cord syndrome (TCS).

**Methods:**

Patients < 17 years who underwent untethering surgery between 2007 and 2018 were screened for eligibility. Those who had undergone primary transection of the FT, and had preoperative radiological findings of fatty filum, thickened FT, or low-lying conus, below the pedicles of L2, were included. The cohort was divided into symptomatic and asymptomatic depending on clinical presentation. Surgical complications and functional outcome was recorded.

**Results:**

In total, 95 patients were included, of whom 62 were symptomatic. In symptomatic patients, the main indications for radiological evaluation were scoliosis (29%) and motor symptoms (19%). In asymptomatic patients, skin stigmata (76%) were the most common finding. Fatty or thick FT was the most common radiographic finding, seen in 61% of symptomatic and 79% of asymptomatic cases. All patients underwent transection of the FT and were followed for a median of 1.8 years. A postoperative complication occurred in 12%, all Ibanez type Ib and managed without invasive treatment. For the symptomatic cohort, significant improvement was seen for both urodynamic assessment (48% improved, *p* = 0.002) and sensorimotor function (42% improved, *p* < 0.001).

**Conclusions:**

Neurological improvement or halted deterioration was seen in the majority of symptomatic cases. Asymptomatic patients did not experience any severe complications. Filum transection should be offered to symptomatic and asymptomatic patients upon diagnosis of fatty filum, thickened FT, or low-lying conus.

## Introduction

### Embryology

The development of the conus medullaris, cauda equina, and filum terminale (FT) differs from that of the spinal cord. While the development of the spinal elements above the level of S2 is referred to as primary neurulation, involving the formation of the neural tube, the spinal elements below S2 are formed through a process called canalization where the neural tube is extended beyond the posterior neuropore [15]. Here, undifferentiated cells form the caudal cell mass and subsequently create a tubular structure which unites with the inferior neural tube to form the conus medullaris, cauda equina, and filum terminale (FT). As the vertebral column grows more than the spinal cord, the conus ascends relative to the vertebrae, and the filum elongates. At 2 months of age, the conus has reached the adult level at the L1/L2 disc [24].

Fat in the FT may represent mesodermal cells that did not properly migrate to their normal position in the process of canalization. Some studies suggest that it is the fatty infiltration of the filum itself that causes tethering and correlates the amount of fat infiltration to the degree of symptoms [6, 20]. However, whether the fat itself alters the elastic properties of the filum or if it simply constitutes radiological evidence of a malformed filum remains unclear [2, 24].

### Tethered cord syndrome and fatty filum terminale

It is believed that the normal FT is a viscoelastic band that anchors, stabilizes, and buffers the distal spinal cord from traction [4]. A lack of elasticity in the FT has been described to be important for the development of tethered cord syndrome (TCS) [7]. TCS is defined as the clinical manifestation of an abnormal traction within the distal spinal cord, with a secondary compromise of blood supply and metabolism [16, 18, 22, 24]. Reportedly, the degree of traction on the conus determines the clinical picture of TCS, meaning that significant traction causes an earlier and more severe presentation of symptoms [22]. The syndrome is associated with motor and sensory dysfunction in the lower limbs, bladder and bowel disturbances, scoliosis, back pain, and foot deformities [5, 11, 14, 24]. The prevalence of fatty filum has been reported to range from 0.24 to 5% in children [3, 6, 12, 18, 20, 21].

Discreet anomalies in the lumbosacral region, such as sacral and coccygeal pits, deviations of the crena ani and naevi are associated with an increased prevalence of fatty filum, and, conversely, skin stigmata appear in 86–90% of all spinal lipomas [20, 21]. In large series of lumbosacral lipomas causing TCS, fatty filum constitutes approximately 13–26% [2]. Several authors advocate a routine MRI for children with perineal malformations such as anal atresia and Currarino syndrome [19–21].

Radiological confirmation of a TCS diagnosis includes identification of a low-lying conus, fatty filum, or a thickened FT (> 2 mm) and normally requires MRI, even if ultrasound may provide diagnostic imaging in infants of less than 6 months of age [22].

For patients with clinical symptoms and radiological findings of TCS, data favors surgical de-tethering. For patients exhibiting only radiological findings of fatty filum or low-lying conus without evident clinical symptoms, the options are prophylactic surgery or expectancy. However, the ideal management strategy is elusive since the natural history of TCS is not fully understood [8].

The aim of this study was to perform a population-based retrospective study on a surgically treated pediatric cohort with radiological signs of TCS to evaluate the surgical outcomes and clarify possible benefits and risks of filum terminale transection.

## Material and methods

### Patient selection

The study hospital is a publicly funded and owned tertiary care center serving a region of roughly 2.3 million inhabitants and the only pediatric neurosurgical center in the region. The surgery management software Orbit (Evry Healthcare Systems, Solna, Sweden) was used to identify the patients. Patient records and imaging data from digital hospital charts were retrospectively reviewed using the medical record software TakeCare (CompuGroup Medical Sweden AB, Farsta, Sweden). The study was approved by the regional ethical review board (2018/1873–31), who waived the need for informed consent.

**Fig. 1:**
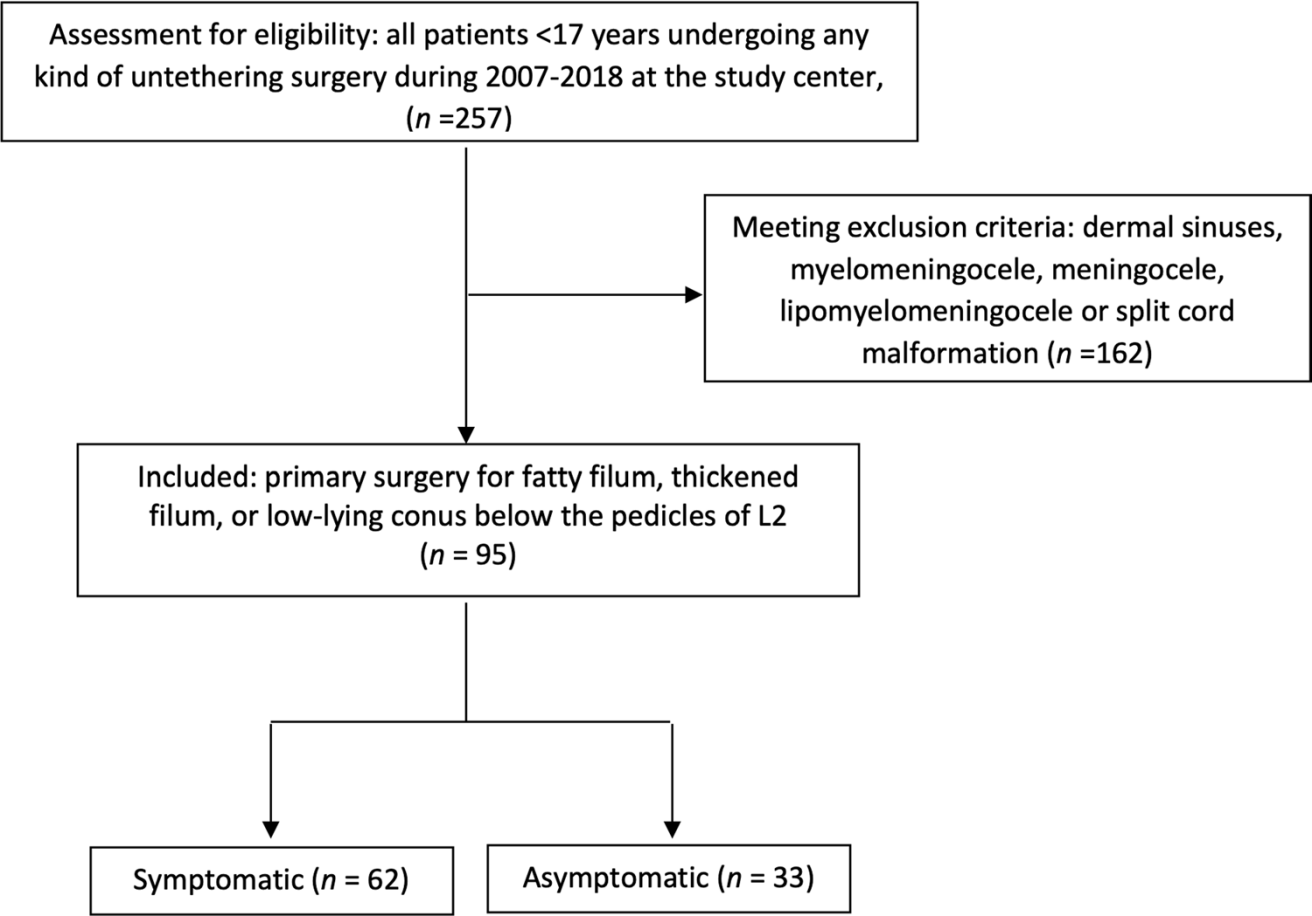
Flowchart of the patient inclusion process

All children below the age of 17 that underwent any kind of untethering procedure (*n* = 257) between the years 2007 and 2018 were screened. Patients with fatty filum, thickened FT, or low-lying conus (below the pedicles of L2), who had undergone primary untethering surgery, were eligible for inclusion (*n* = 95). Patients with dermal sinuses, myelomeningocele, meningocele, lipomyelomeningocele, or split cord malformation (*n* = 162) were excluded (Fig. 1). The FT was defined as thickened when its diameter exceeded 2 mm on a T1-weighted MRI [3, 12, 20]. The level of the conus medullaris in relation to the vertebral bodies was determined by two pediatric neuroradiologists based on T2-weighted axial and sagittal images.

According to regional guidelines and clinical routine, all children with symptoms or findings suggestive of TCS are referred to the study center for neuropediatric evaluation. Conditions prompting MRI included sacral pits above the level of the coccyx, deviation of the crena ani, hair tufts, hypo- or hyperpigmentation of the skin, or any combination of the above findings. Similarly, the findings of midline anomalies and congenital syndromes, such as esophageal atresia, anal atresia, Currarino syndrome, VACTERL syndrome, or heart and kidney malformations associated with midline malformations, prompted an MRI investigation. In addition, patients with juvenile scoliosis not responding to conservative treatment are investigated with MRI. Surgery is offered to all patients fulfilling radiological criteria of TCS, irrespective of symptoms.

All included patients underwent preoperative evaluation by a team composed of a pediatric neurologist, a neurosurgeon, a nurse specialized in urodynamic evaluation, and a physiotherapist.

The urodynamic assessment serves to identify signs of neurogenic bladder dysfunction, and the physiotherapy assessments include testing and observation for sensory or motor impairments (summarized as sensorimotor function) and pain. In this study, the outcome variables from these preoperative assessments are dichotomized into normal or impaired.

**Fig. 2:**
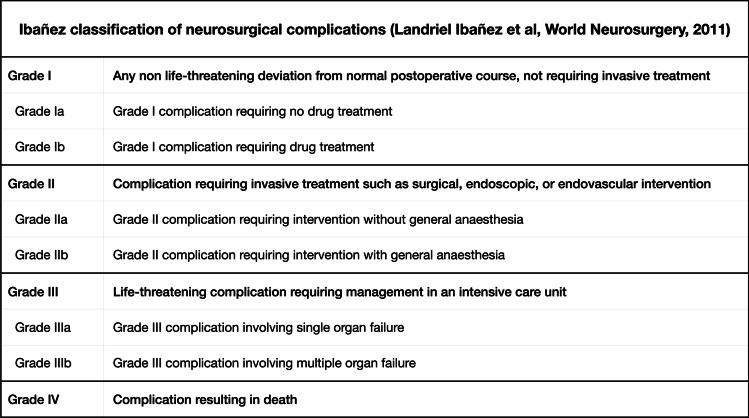
Ibañez classification of neurosurgical complications

The study cohort was divided into two groups, symptomatic or asymptomatic, depending on whether preoperative symptoms attributable to TCS were present or not. In this context, scoliosis is a possible result of TCS, and these patients were allocated to the symptomatic group. All surgical complications were recorded systematically according to the Landriel Ibanez classification for neurosurgical complications (Fig. 2) [10].

### Surgical technique

All surgeries were performed by either of three attending pediatric neurosurgeons. Patients were routinely administered a single prophylactic dose of cefuroxime (50 mg/Kg) or clindamycin (25 mg/Kg) in the case of known penicillin allergy. With the patient in the prone position, a posterior midline approach was used. A single level laminotomy was performed at L4, below the conus. In most cases, an ultrasonic bone scalpel was used (Misonix Inc., Farmingdale, NY, USA).

Under the microscope, the dura was incised in the midline and was held open by sutures. The arachnoid was dissected sharply, and the FT was identified visually and divided after coagulation. In all cases, watertight dural closure was performed, and the lamina reinstated using resorbable sutures in the supraspinal ligaments and thoracolumbar fascia. The surgical wound was closed in layers. No intraoperative neurophysiological monitoring was used. Postoperative management included a tapered protocol of intravenous analgesics (morphine, clonidine, paracetamol). In addition, all patients were kept at flat bed rest for 48 h to reduce the risk of CSF leakage.

### Postoperative evaluation

Complication data (within 30 days), including wound infection, meningitis, CSF-leakage, and urinary tract infection, were graded according to Landriel Ibanez [10]. Three months postoperatively, all patients underwent a renewed team assessment at the outpatient clinic. Urodynamic and physiotherapy assessments were repeated 6 months after the surgery and compared to preoperative values and data are presented as normal or impaired.

### Statistics

The Shapiro–Wilk test was used to evaluate the normality of continuous data. As all continuous data significantly deviated from a normal distribution pattern (Shapiro–Wilk test *p* value < 0.05), it is presented as median (interquartile range) and categorical data is presented as numbers (proportion). Baseline, treatment, and outcome data, stratified by clinical presentation (symptomatic vs. asymptomatic patients), were compared using the Mann Whitney *U* test (continuous non-parametric data), the Chi2 test (categorical data), or Fisher’s exact test (categorical data with sample size ≤ 5). The Wilcoxon matched-pairs signed-ranks test was used to determine if surgery was associated with significant improvement in bladder and sensorimotor function. Univariable regression models were employed to determine possible predictors of improved bladder and sensorimotor function. All analyses were conducted using the statistical software program R version 4.0.5. Statistical significance was set at *p* < 0.05.

## Results

### Baseline data and clinical presentation

Ninety-five patients were included in the study. The median age was 3.5 years, and 48 (51%) were female. Midline abnormalities were seen in 45 (47%), fatty filum in 64 (67%), and low-lying conus (below L2) in 48 (51%). Sixty-two (65%) patients were symptomatic, where motor deficit (*n* = 43, 74%) was the most common presenting symptom, followed by scoliosis (*n* = 33, 57%) and bladder dysfunction (*n* = 25, 44%). Symptomatic patients were significantly older than asymptomatic patients (6.3 vs. 1.8 years, *p* < 0.001), but no other differences in baseline data were observed (Table 1).

**Table 1:** Baseline data and clinical presentation

Variable	All patients (*n* = 95)	Asymptomatic (*n* = 33)	Symptomatic (*n* = 62)	*p* value
Female sex	48 (51%)	16 (48%)	32 (52%)	0.772
Age (years)	3.5 (1.7–7.9)	1.8 (1.1–2.3)	6.3 (2.5–11.0)	** < 0.001**
Symptoms				
Pain	21 (23%) (5 missing)	-	21 (37%) (5 missing)	-
Bladder dysfunction	25 (28%) (5 missing)	-	25 (44%) (5 missing)	-
GI dysfunction	24 (27%) (6 missing)	-	24 (43%) (6 missing)	-
Scoliosis	33 (36%) (4 missing)	-	33 (57%) (4 missing)	-
LE deformity	20 (22%) (4 missing)	-	20 (34%) (4 missing)	-
Motor deficit	43 (47%) (4 missing)	-	43 (74%) (4 missing)	-
Sensory deficit	12 (14%) (7 missing)	-	12 (22%) (7 missing)	-
Development delay	17 (19%) (4 missing)	-	17 (29%) (4 missing)	-
Midline abnormality	45 (47%)	14 (42%)	31 (50%)	0.481
Filum lipoma	9 (9.5%)	4 (12%)	5 (8.1%)	0.715
Fatty filum	64 (67%)	26 (79%)	38 (61%)	0.083
Conus below L2	48 (51%)	17 (52%)	31 (50%)	0.888
Syringohydromyelia	6 (6%)	2 (6%)	4 (6%)	> 0.999

Data presented as median (IQR) or *n* (%). Bold text in the *p* value column indicates a statistically significant association (p < 0.05). Abbreviations: *GI* gastrointestinal; *LE* lower extremity

### Treatment and complications

Filum transection was performed in all patients. The median operating room (OR) time was 81 min. A postoperative complication occurred in 11 (12%) patients, all of which were classified as Ibanez type Ib and thus managed without invasive treatment. Median hospital stay and follow-up time was 4.0 days and 1.8 years, respectively. Compared to the asymptomatic cohort, symptomatic patients showed significantly longer OR time (87 vs. 66 min, *p* = 0.002) and longer hospital stay (5.0 vs. 4.0 days, *p* = 0.010) and had a longer follow-up time (2.5 vs. 0.8 years, *p* < 0.001) (Table 2).

**Table 2:** Treatment and complications

Variable	All patients (*n* = 95)	Asymptomatic (*n* = 33)	Symptomatic (*n* = 62)	*p* value
OR time	81 (64–107)	66 (59–84)	87 (72–123)	**0.002**
Filum transection	95 (100%)	33 (100%)	62 (100%)	> 0.999
Post-op complication	11 (12%) (2 missing)	4 (12%)	7 (12%) (2 missing)	> 0.999
CSF-leak, no surgery	1 (1.1%)	0 (0%)	1 (1.6%)	> 0.999
Antibiotics for wound infection	6 (5.6%)	3 (9.1%)	3 (5.0%)	0.415
Antibiotics for other infections	3 (4.2%)	0 (0%)	3 (4.8%)	0.549
Calicivirus infection	1 (1.1%)	1 (3.0%)	0 (0%)	0.347
Reoperation	0 (0%)	0 (0%)	0 (0%)	> 0.999
Readmission	3 (3.2%)	1 (3.0%)	2 (3.2%)	> 0.999
Hospital stay	4.0 (4.0–5.0)	4.0 (4.0–5.0)	5.0 (4.0–5.0)	**0.010**
Follow-up time (years)	1.8 (0.8–3.8) (1 missing)	0.8 (0.6–2.5)	2.5 (1.2–4.2) (1 missing)	** < 0.001**

Data presented as median (IQR) or *n* (%). Bold text in the *p* value column indicates a statistically significant association (*p* < 0.05). Abbreviations: *CSF* cerebrospinal fluid; *OR* operating room

### Functional outcome

For the symptomatic cohort, surgery was associated with a significant improvement in both bladder (*p* = 0.002) and sensorimotor function (*p* < 0.001), with 48% showing improvement in bladder function and 42% improvement in sensorimotor function (Table 3). No significant preoperative predictors of improved functional outcome were identified (Tables 4 and 5).

**Table 3:** Change in neurological status

	Bladder function	Sensorimotor function
**Patients with preoperative deficit (*n*)**	**25**	**43**
Improved	12 (48%)	18 (42%)
Unchanged	7 (28%)	17 (40%)
Worse (increased deficit)	1 (4.0%)	0 (0%)
Missing	5 (20%)	8 (19%)
*p* value (paired testing)	**0.002**	** < 0.001**

Data presented as *n* (%). Bold text in the *p* value column indicates a statistically significant association (*p* < 0.05)

**Table 4:** Univariate logistic regression predicting improvement in urodynamic function

Variable	*p* value	OR (95% CI)
Male sex	0.365	0.43 (0.06–2.60)
Age (years)	0.771	1.03 (0.82–1.32)
Midline abnormality	0.187	0.20 (0.01–1.68)
Fatty filum	0.079	6.00 (0.91–56.3)
Conus below L2	0.153	0.24 (0.03–1.54)
Ibanez Ib complication	0.762	0.64 (0.02–17.9)

Data presented as median (range) or count (proportion). Bold text indicates a statistically significant correlation (*p* < 0.05)

**Table 5:** Univariate logistic regression predicting improvement in sensorimotor function

Variable	*p* value	OR (95% CI)
Male sex	0.862	1.12 (0.30–4.32)
Age (years)	0.320	0.92 (0.77–1.08)
Midline abnormality	0.601	0.70 (0.18–2.66)
Fatty filum	0.632	1.40 (0.35–5.71)
Conus below L2	0.626	0.72 (0.18–2.75)
Ibanez Ib complication	0.587	2.00 (0.17–45.6)

Data presented as median (range) or count (proportion)

## Discussion

### Signs and symptoms in TCS

In TCS, the development of symptoms is alarming, since deficits may be irreversible despite rapid surgical intervention [18]. TCS is associated with lower limb pain and weakness, fecal and urinary incontinence, back pain, scoliosis, and foot deformities [1, 2, 7, 22]. In this study, scoliosis was evident in 36% of the cases, reflecting the importance of screening for TCS in idiopathic scoliosis.

Symptoms of bladder dysfunction are late manifestation of TCS and are considered early signs of permanent damage [4, 22]. Motor symptoms are more prevalent than sensory deficits, and children may present with delayed gait development, spasticity, hyper- or hyporeflexia, and muscular atrophy [4]. In this study, symptoms of bladder dysfunction were seen in 44% of the symptomatic group and ranged from obvious incontinence to subtle, subclinical signs only seen on urodynamic assessment. Sensorimotor symptoms were present in 74% of the symptomatic patients; all patients with sensory deficits had a simultaneous motor deficit. These findings are in accordance with previously published reports [13].

### Surgical outcome

The primary goal of surgery in TCS is neurological improvement or halted deterioration. However, early intervention also aims to improve the long-term neurological function [4, 11]. Transection of FT has been shown to halt the progression of scoliosis, improve pain, and reduce daytime urinary incontinence [11, 22]. In this study, significant changes were seen in the symptomatic group where 48% and 42% improved after surgery regarding bladder and sensorimotor functions, respectively.

When operating asymptomatic patients, the goal is to prevent the development of future neurological symptoms or impairments that may be attributed to TCS. To justify prophylactic surgery, it is important to show that the procedure is safe with no or few complications.

The incidence of neurological injury due to transection of the FT is reportedly less than 1% [4]. In general, complications are infrequent but include infection, nerve root injury, or CSF leak. Bhimani et al. reported a superficial wound infection rate of 5% in a series of 3682 pediatric patients undergoing the procedure [1]. Our reported rate of 5.6% is well in line with that result. However, another 3 patients were treated for possible superficial infections within 30 days of surgery without a positive culture. Since the surgery is offered as a low-risk procedure to small children, it arguably lowers the threshold to start antibiotics for any sign suggestive of infection. No cases of nerve root damage, hematoma, or pseudomeningocele were seen. One of the patients in the symptomatic group was readmitted to hospital within 30 days due to CSF leakage that was managed conservatively with bed rest. Although rare, re-tethering may occur after simple filum sectioning [18]. However, no cases of re-tethering were seen in our material during the follow-up.

### What is the role for prophylactic surgery in TCS?

Published data on surgery in TCS show that early surgical intervention alleviates traction on the spinal cord and prevents the development of irreversible neurological damage [21, 22]. In general, early surgical intervention is associated with improved outcome [11]. Moreover, neurologic deficits reportedly stabilize after surgery without further decline in 80 to 90% of cases [11]. However, available data regarding the development of neurological decline in asymptomatic patients with radiological signs of TCS are conflicting. Some studies have reported a conversion to symptomatic TCS at a rate of 3–4% per year [9, 14, 17, 23]. Other studies find no significant differences or only non-significant tendencies between the surgically and conservatively treated groups, despite long follow-up times [9, 17]. In contrast, Wykes et al. found that 29% of the conservatively managed cases became symptomatic within a follow-up time of 5.9 years [22]. Transection of the filum terminale is considered a low-risk procedure [1, 4, 7, 20], and based on the potentially irreversible and lifelong neurological impairments associated with TCS, prophylactic surgery has been advocated. Nonetheless, if prophylactic surgery for TCS can prevent the development of future neurological deficits is a matter of controversy [1].

Adding to the complexity, symptoms of TCS may progress slowly, mimicking a lagging normal development, and become clinically overt at a much later time point. Notably, a slight postoperative improvement in urodynamic and sensorimotor assessments, compared to the preoperative data, was noted in a few asymptomatic patients. This may be explained by the difficulty in assessing what is impaired in the preoperative work-up of a pediatric population. Alternatively, it may reflect normal neurological development taking place during the postoperative period prior to follow-up.

### Limitations

There is currently no class I evidence regarding TCS and prophylactic surgery. Most studies, such as this one, are retrospective and lack uniformity. The retrospective nature of this study and the rather small sample size of our cohort make statistical evaluation difficult. Patients were retrospectively identified based on having undergone a spinal cord untethering procedure. Thus, this study does not contain any data from patients where surgery was declined, and the choice was made to monitor the patients with clinical and radiological examinations.

## Conclusions

Whether prophylactic surgery for TCS can prevent the development of future neurological deficits is a matter of controversy. This study shows that surgery for TCS in a pediatric population is safe with few complications and results in significant improvement in bladder and sensorimotor function when symptoms are present. Since conservative treatment may put the patient at risk for neurological impairment, we recommend a liberal attitude towards surgery for TCS, based on radiological findings and irrespective of preoperative symptoms.

## Data Availability

The datasets used and/or analyzed during the current study are available from the corresponding author on reasonable request.
